# Miscellaney

**Published:** 1896-09

**Authors:** 


### Miscellany.

---

To Drive Away Flies.—Dr. H. S. Baketel, of Derry, N. H., writes: Many practitioners of medicine among the poorer classes are greatly annoyed by flies in the sick room. The annoyance to the patient is doubly great. Such, at least, was my experience not long since on New York's great east side. An excellent safeguard against these pests is the sweet-pea flower. The Lathrus maritimus, the purple variety, grows near the seacoast from New Jersey around to Oregon, and beside the coasts of the Great Lakes. The Lathyrus ochroleucus is found on the hillsides from New England to Minnesota, and even further west. It is distinguished by its small, yellowish-white flower. Either of these varieties can be grown in the sick room, and the sweet odor emanated seems very offensive to the ordinary house fly.—*Chicago Medical Record.*

---

An advertising specialist of Cincinnati, Dr. W. I. Kelley, has been arrested and fined $50 by the Kentucky courts, upon a warrant sworn out by Dr. J. M. Mathews, of Louisville, the president of the State Board of Health, charging him with practising medicine without a state license. It is alleged that Dr. Kelley applied for a license, which was refused him. The Hon. W. C. P. Breckenridge defended him, and has given notice of an appeal.—*Atlantic Medical Weekly.*

---

The First Railway Accident.—From an historical standpoint the Baltimore & Ohio railway is preëminently interesting. It was on this railroad that the first American locomotive ever constructed was put into operation. History tells us that Peter Cooper was the master mechanic who conducted its construction, and that on August 28th, nearly sixty-six years ago, he drew with it the first passenger car ever propelled by steam on any railroad in the world. On this historic occasion Mr. Cooper was not only the master mechanic but engineer, and in addition to having gained for him the distinction of having built the first railway engine in America and drawn the first passenger car in the world, he on the same occasion was the subject of an accident, in which, the record tells us, he severely jammed his thumb, and which is believed to be the first railway casualty in the history of the world.—*Columbus Medical Journal.*

---

Physicians Should Work Less.—Dr. Kortright, in the *Brooklyn Medical Journal*, says that arterial sclerosis is a common cause of death in physicians. The lesson that we should learn from our deceased colleagues, he states, is not to work too long. When you find your arterial tension increasing, your temporal artery becoming tortuous, your radial growing hard, especially if you have a little palpitation and pass an increased amount of limpid urine, whatever your years, know that old age is upon you. Henceforth shape your life like one that is old. Curb your ambition. Be content with a small practice. Reduce your expenses. Give up your night work. Decline confinements. Take a long vacation in summer. Retire early. Eat abstemiously. Drink not at all. Sell your horse. Take a great deal of moderate exercise in the open air. Watch the functions of the skin. Guard against a chill. Cultivate an even disposition. Study to be quiet.—*Maryland Medical Journal.*

---

The City of Cleveland has enacted a city ordinance that fixes the license law tax at $300 a year for astrologists, fortune-tellers and seers.

---

The new drug company, established under the sanction and encouragement of the State Pharmaceutical Association, is getting into shape for active operations. Headquarters have been secured in the thirteen-story Guaranty building. The incorporators are Messrs. Stoddart, Peterson, McArthur, Smither, Gregory, McEachren, Hosmer, Perkins, Liebetrut, Reiman, Tilma, Anthony, Hayes, Lockie, Cradduck, Kreuz, Kaestner, Rogers and Lustig, of Buffalo; Dalton, of Syracuse; Schnell, of Binghamton; and Osmun, of New York. C. O. Rano has been mentioned in connection with the secretaryship and general management.—*Buffalo Druggist.*

---

The liability to prosecution for damages in abdominal surgery was recently discussed in the *New York Medical Journal* by Dr. Cyrus A. Kirkley, of Toledo. He said that the average juryman looked upon the medical expert as a witness having a bias for the side calling him. After setting forth the reasons for a reform in the matter of medical expert testimony, he commended the plan adopted in England, of having all the medical witnesses of both sides confer together before giving their testimony, and suggested that if the court were to appoint a medical commission to hear and pass upon the medical points involved, some of the present abuses would be avoided. He also quoted a legal opinion to the effect that a surgeon, operating in an emergency by abdominal section upon a patient who had no chance for life without such operation, should not be liable in damages if this view of the urgency of the case were concurred in by other well-qualified practitioners.—*Medical and Surgical Reporter.*

